# Does prolonged experimental duration influence the interpretation of growth-based arginine requirement estimates in Japanese quails?

**DOI:** 10.1016/j.psj.2026.107655

**Published:** 2026-08-20

**Authors:** Mehran Mehri

**Affiliations:** Department of Animal Sciences, Faculty of Agriculture, University of Zabol; Sistan, Iran,

I read with considerable interest the article by Ghiasvand et al. (2025) investigating the effects of standardized ileal digestible (SID) arginine (Arg) on growth performance, intestinal morphology, and hepatic expression of growth-related genes in Japanese quails.

I would like to raise a methodological concern regarding the interpretation of the reported SID Arg requirement. A distinguishing feature of this study is its 35-day continuous dose–response bioassay, in which quails were maintained from hatch to 35 days of age on diets containing 0.75 to 1.75% SID Arg (Ghiasvand et al., 2025). Requirement estimates were subsequently derived from growth performance using broken-line and quadratic regression models. Although these approaches are well established for nutrient requirement estimation (Robbins et al., 2006), their biological interpretation assumes that the measured response remains physiologically comparable throughout the experimental period.

This assumption deserves careful consideration in long-term amino acid bioassays. After 35 days of continuous exposure to a severely Arg-deficient diet (0.75% SID Arg), growth performance may no longer represent only dietary Arg supply but also cumulative physiological responses to prolonged nutrient restriction. Consistent with this possibility, Arg-deficient birds exhibited not only depressed growth but also significant alterations in jejunal morphology and hepatic expression of mTOR, RPS6KA1, HSP70, and HSP90 (Ghiasvand et al., 2025), indicating that prolonged deficiency modified multiple physiological systems beyond growth alone. The molecular findings further highlight this concern. Broken-line regression identified Arg requirements of 1.19% for average daily gain and 1.30% for feed conversion ratio, whereas hepatic TOR and RPS6KA1 expression reached their highest levels at 1.50% and 1.75% SID Arg, respectively (Ghiasvand et al., 2025). Because these genes are central regulators of mTOR-mediated protein synthesis and are responsive to Arg availability (Yuan et al., 2015), this divergence between molecular and performance endpoints deserves further discussion. If anabolic signaling continues to respond beyond the dietary concentration identified by growth performance, *can growth measured after a prolonged bioassay alone adequately define physiological Arg adequacy?*

I respectfully suggest that growth-derived SID Arg requirements from long-term bioassays should be interpreted cautiously until it is established that prolonged dietary deficiency does not alter the biological response used for requirement estimation, especially when the estimated Arg requirements were based on broiler-derived SID coefficients because amino acid digestibility may differ between species owing to differences in gastrointestinal development, digestive physiology, and nutrient utilization. Therefore, both the applicability of broiler-derived SID coefficients to Japanese quails and the biological interpretation of SID Arg requirement estimates warrant further validation.

## Disclosures

No conflict
